# Absorbability, Mechanism and Structure-Property Relationship of Three Phenolic Acids from the Flowers of *Trollius chinensis*

**DOI:** 10.3390/molecules191118129

**Published:** 2014-11-05

**Authors:** Xiu-Wen Wu, Ru-Feng Wang, Li-Jia Liu, Li-Na Guo, Can Zhao

**Affiliations:** School of Chinese Materia Medica, Beijing University of Chinese Medicine, Beijing 100102, China; E-Mails: wuxiuwen0725@126.com (X.-W.W.); lily199109231@163.com (L.-J.L.); gngn2010@163.com (L.-N.G.); 15201391380@163.com (C.Z.)

**Keywords:** *Trollius chinensis*, Ranunculaceae, phenolic acids, absorbability, Caco-2 cell monolayer model

## Abstract

The absorption properties, mechanism of action, and structure-property relationship of three phenolic acids isolated from the flowers of *Trollius chinensis Bunge*, namely, proglobeflowery acid (PA), globeflowery acid (GA) and trolloside (TS), were investigated using the human Caco-2 cell monolayer model. The results showed that these three phenolic acids were transported across the Caco-2 cell monolayer in a time and concentration dependent manner at the P_app_ level of 10^−5^ cm/s, and their extent of absorption correlated with their polarity and molecular weight. In conclusion, all three of these compounds were easily absorbed through passive diffusion, which implied their high bioavailability and significant contribution to the effectiveness of *T. chinensis.*

## 1. Introduction

**Figure 1:**
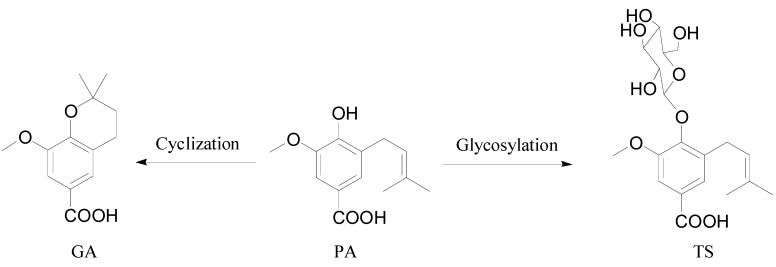
Chemical structures of PA, GA and TS.

Proglobeflowery acid (systematic name: 4-hydroxy-3-methoxy-5-(3-methylbut-2-en-1-yl)benzoic acid) (PA), globeflowery acid (systematic name: 8-methoxy-2,2-dimethylchroman-6-carboxylic acid) (GA) and trolloside (systematic name: 3-methoxy-5-(3-methylbut-2-en-1-yl)-4-(((2*S*,3*R*,4*S*,5*S*,6*R*)-3,4,5-trihydroxy-6-(hydroxymethyl)tetrahydro-2*H*-pyran-2-yl)oxy)benzoic acid) (TS) are three important phenolic acids isolated from the flowers of *Trollius chinensis* Bunge which mainly contain phenolic acids, flavonoids and alkaloids [1,2,3]. Structurally, these three phenolic acids are *p*-hydroxy acid derivatives. PA and GA are isomers, the former being intramolecularly cyclized into the latter under acidic condition, while TS is the glycosylated form of PA (Figure 1). Pharmacological studies have demonstrated that phenolic acids, including these three compounds, exhibit various biological activities, which are closely related to the effect of these flowers in the treatment of respiratory infections, pharyngitis, tonsillitis and bronchitis. For example, PA showed inhibitory activity against *Pseudomonas aeruginosa* and *Staphylococcus aureus* with MIC values of 16 and 200 mg/L, respectively [4,5], and also had weak antiviral activity against Para 3 with an IC_50_ value of 184.2 μg/mL [6]. GA exhibited significant antiviral activity against influenza A with an IC_50_ value of 42.1 μg/mL, and TS displayed moderate inhibitory effect against *Streptococcus pneumonia* with a MIC value of 128 mg/L [7]. Thus, these three phenolic acids can be considered as the bioactive components of the flowers of *T. chinensis.* However, to what extent they are effective in the human body depends on their bioavailability and metabolism *in vivo*. As is well known, absorbability is a crucial factor which influences the bioavailability of drugs, especially of those being administrated orally. For this reason, the present study investigated the absorption property and mechanism, as well as structure-absorbability relationship of these three phenolic acids using the well-recognized human Caco-2 monolayer model, so as to predict their bioavailability and understand their contribution to the effectiveness of these flowers.

## 2. Results and Discussion

### 2.1. Results

#### 2.1.1. Validation of HPLC Analytical Method

**Figure 2:**
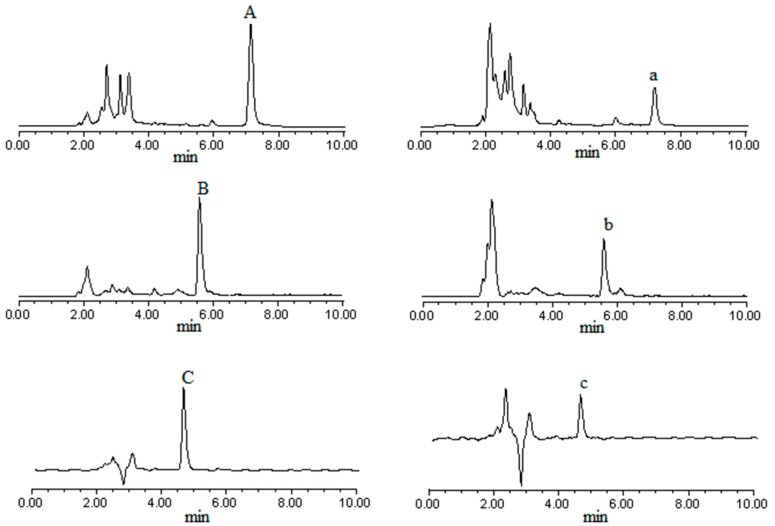
HPLC chromatograms of PA, GA and TS.

Notes: A, B, C were chromatograms of reference solutions of PA, GA and TS; a, b and c were chromatograms of sample solutions of PA, GA and TS across the monolayer.

The HPLC chromatograms of the analytes (Figure 2) showed that all chromatographic peaks of interest were well resolved at baseline under the optimal conditions. All these analytes were quantified by linear regression analysis, and their contents were all within the linear limits. The RSD values for intra-day and inter-day precision and stability tests were all well below 2.3%, indicating that the analytical method employed was validated and the samples were stable under the test condition.

#### 2.1.2. Validation of Caco-2 Monolayer Model

The TEER values of the monolayers developed in this study increased steadily over time and reached over 600 Ω∙cm^2^ on day 21 after seeding. The P_app_ values of reference controls, propranolol and atenolol, tested with the monolayers in this study were (1.24 ± 0.10) × 10^−5^ cm/s and (6.92 ± 0.51) × 10^−7^ cm/s respectively, which were in good agreement with the acceptable values reported in the literature [8]. Thus, the Caco-2 cell monolayer model was validated for assessment of the intestinal absorption potential of the compounds of interest.

#### 2.1.3. Non-Toxic Dose Range of Test Compounds

The survival rates of Caco-2 cells treated with three phenolic acids at different concentrations in MTT assay (Table 1) was all above 95% and no cytotoxicity was observed, whereas those of the Caco-2 cells treated with paclitaxel (the positive control) were below 70% and exhibited dose dependence. These results showed that the Caco-2 cells were not influenced by the test compounds at the concentrations up to 100 μmol/L. Therefore, 25, 50 and 100 μmol/L were chosen as the test concentrations.

**Table 1:** Survival rates of Caco-2 cells treated with test compounds (n = 4).

Compounds	Survival Rate: %		
	25 μmol/L	50 μmol/L	100 μmol/L
PA	118.8 ± 11.6	110.5 ± 3.8	124.5 ± 2.2
GA	107.1 ± 5.7	118.9 ± 13.7	123.6 ± 6.2
TS	98.9 ± 4.3	107.3 ± 7.0	118.2 ± 8.1
Paclitaxel	67.3 ± 8.2	45.5 ± 6.7	37.4 ± 3.5

#### 2.1.4. Transport of Test Compounds

Under the conditions of this experiment, the human intestinal permeability of PA, GA and TS was evaluated using Caco-2 cell monolayer model. The transport was monitored for a period of 90 min. The bidirectional, *i.e.*, both from apical (AP) to basolateral (BL) side and from BL to AP side apparent permeability coefficients (P_app_) values of PA, GA and TS (Table 2) were all at the level of 10^−5^ cm/s.

**Table 2:** P_app_ values of PA, GA and TS obtained with Caco-2 model.

Concentration (μmol/L)		P_app AP→BL_ (×10^−5^ cm/s)	P_app BL→AP_ (×10^−5^ cm/s)	P_ratio_ (P_app AP→BL_/P_app BL→AP_)
PA	25	3.17 ± 0.04	2.54 ± 0.22	1.25
	50	2.80 ± 0.13	2.56 ± 0.19	1.1
	100	2.96 ± 0.11	2.59 ± 0.14	1.14
	average	2.98 ± 0.13	2.56 ± 0.18	1.16
GA	25	2.14 ± 0.20	2.21 ± 0.08	0.96
	50	1.59 ± 0.02	2.00 ± 0.07	0.8
	100	1.70 ± 0.12	1.94 ± 0.10	0.88
	average	1.81 ± 0.11	2.05 ± 0.08	0.88
TS	25	0.86 ± 0.16	0.86 ± 0.04	1
	50	1.06 ± 0.03	1.08 ± 0.06	0.98
	100	0.77 ± 0.09	0.80 ± 0.03	0.96
	average	0.89 ± 0.09	0.91 ± 0.04	0.98

Notes: P_app AP→BL_: transport of test compounds from AP to BL; P_app BL→AP_: transport of test compounds from BL to AP; P_ratio_ (P_app AP→BL_/P_app BL→AP_): the ratio of P_app AP→BL_ to P_app BL→AP_. All data was expressed as the mean ± SD (n = 3). The incubation time was up to 90 min.

#### 2.1.5. Time Course

**Figure 3:**
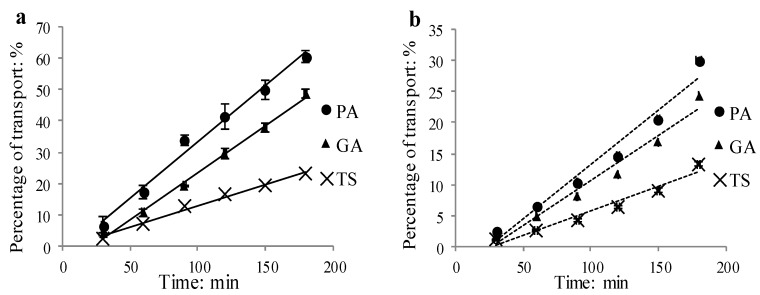
Transport of three phenolic acids across Caco-2 cell monolayer as the function of time at 50 μmol/L (**a**) AP to BL side; (**b**) BL to AP side (means ± SD, n = 3).

The approximate kinetic curves of these three compounds over time (30–180 min, Figure 3) showed that their percentage of transport increased with the incubation time in an approximate linear manner.

#### 2.1.6. Concentration Dependence

**Figure 4:**
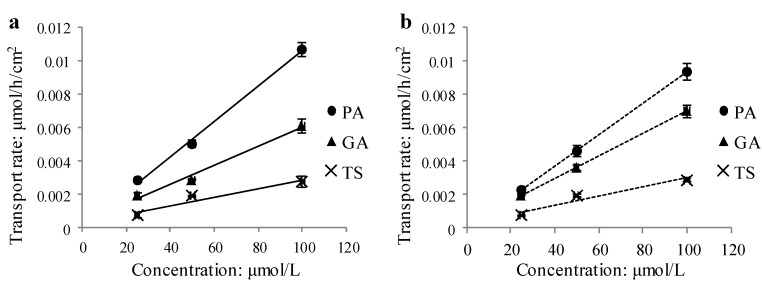
Transport of three phenolic acids across Caco-2 monolayer as the function of concentration at 90 min (**a**) AP to BL side; (**b**) BL to AP side (means ± SD, n = 3).

The kinetic curves of these three compounds over concentration (25–100 μmol/L, Figure 4) showed that their bidirectional transport rate increased with concentration in an approximately linear manner.

#### 2.1.7. Molecular Properties

The molecular properties of PA, GA and TS calculated by software are listed in the Table 3.

**Table 3:** Molecular properties of PA, GA and TS.

Compound	Molecular Mass	LogP	TPSA	H_d_	H_r_	R	V
PA	236.26	2.67	66.761	2	4	4	0
GA	236.26	2.27	55.767	1	4	2	0
TS	398.4	0.83	145.913	5	9	7	0

Notes: H_d_: the number of hydrogen bond donor; H_r_: the number of hydrogen bond receptor; R: the number of rotatable bond; V: the number of violation of the “five rules”.

### 2.2. Discussion

Under the conditions of the experiments, the human intestinal permeability of PA, GA and TS was evaluated using the Caco-2 cell monolayer model. The P_app_ values of PA, GA and TS were at the same magnitude (10^−5^ cm/s) as that of propranolol, which is often used as a reference compound of high permeability [8,9,10]. This implied that these compounds were easily absorbed by human body through intestinal mucosa. It has been well known that “five rules” (H_d_ < 5; H_r_ < 10; LogP < 5; Molecular mass < 500; passive transport) and “Veber rules” (TPSA < 140 Å^2^; R < 10) [11] are useful to evaluate the absorption of compounds. According to these rules, none of these three phenolic acids violate the “five rules”. Unlike PA and GA, however, TS (TPSA = 146 Å^2^) (Table 3) violates the “Veber rules”. Thus, all of these three compounds were easily absorbed, but TS was the relatively poorest absorbed one among them. As a matter of fact, the absorption extent of these three compounds correlated with their molecular polarity and molecular weight. In general, the larger the molecular polarity and molecular weight of the compound, the more difficult it is absorbed. Undoubtedly, TS is the highest in either polarity or molecular weight. Correspondingly, the bidirectional P_app_ values of this compound were the lowest among those of the three compounds. As for PA and GA, they are isomers with the same molecular weight. In this case, molecular polarity is the crucial factor to determine the absorption order of difficulty. PA is less polar than GA, which is evidenced by the RP-HPLC chromatogram of these compounds (Figure 2) and the LogP values (Table 3). Accordingly, the larger bidirectional P_app_ values of PA (Table 2) indicated that it is easier absorbed than GA. The approximate linear increase of bidirectional transport or transport rate of these three compounds over time and concentration (Figure 3 and Figure 4) demonstrated that they were across the monolayer mainly through passive transcellular diffusion. Furthermore, it was confirmed by the fact that the P_app AP→BL_ to P_app BL→AP_ ratios of these three compounds were almost equal to 1 (Table 2), which indicated that neither efflux nor active transport was involved in their absorption.

On the basis of the literature [8], the test phenolic acids would be absorbed over 70% after oral application *in vivo*. In our previous study, the most concentrated phenolic acid in the flowers of *T. chinensis*, *i.e.*, veratric acid was demonstrated to be well absorbed. All in all, the phenolic acids in the flowers of *T. chinensis* have a good absorbability; thus, these compounds should be attached importance in the evaluation of effective components of these flowers.

## 3. Experimental

### 3.1. General

Dulbecco’s Modified Eagle’s Medium (DMEM), fetal bovine serum (FBS) and trypsin were supplied by Gibco (Grand Island, CA, USA). Nonessential amino acids (NEAA), penicillin and streptomycin were purchased from Corning Costar (Cambridge, MA, USA). 3-(4,5-dimethylthiazolzyl)-2,5-diphenyl tetrazolium bromide (MTT), ethylene diaminetetraacetic acid (EDTA), dimethyl sulfoxide (DMSO), propranolol and atenolol with purity of minimum 98% were products of Sigma Chemical Co. (Deisenhofen, Germany). Proglobeflowery acid, globeflowery acid and trolloside were isolated from the flowers of *T. chinensis* by our research group and their purities were determined to be over 98% by HPLC. Paclitaxel drug product (Batch No. 20140201, Strength 30 mg/5 mL) was provided by Beijing SL Pharmaceutical Co., Ltd. (Beijing, China). Other chemicals were of analytical grade and solvents used in high-performance liquid chromatography (HPLC) were of HPLC grade. Transwell plates (insert diameter 12 mm, pore size 3.0 μm, membrane growth area 1.12 cm^2^) were purchased from Corning Costar.

### 3.2. Plant Material

The flowers of *T. chinensis* were purchased from the Anguo drug market of Hebei Province and identified by Ru-Feng Wang. A voucher specimen (No. 20120526) has been deposited at the Herbarium of School of Chinese Materia Medica, Beijing University of Chinese Medicine.

### 3.3. Extraction and Isolation of GA, PA and TS

About 2 kg of the dried flowers of *T. chinensis* was extracted with 36 L of 95% ethanol under reflux for 2 h. The extract (0.6 kg) was concentrated *in vacuo*, and then was suspended in 1.2 L of water. The suspension was partitioned successively with each 1.2 L of petroleum ether, ethyl acetate and *n*-butanol. The resultant EtOAc-soluble part (50 g) was separated by polyamide (30~60 mesh) column chromatography eluted with H_2_O, 20% MeOH, 50% MeOH, 70% MeOH and 95% MeOH, respectively, to afford five major fractions (Fr.A-Fr.E). Fr.C (2.1 g) was subjected to silica gel chromatography eluted with gradient CHCl_3_–MeOH from 30:1 to 1:1 to give five fractions (Fr.C1~Fr.C5). Fr.C2 (310 mg) was further isolated with silica gel column chromatography eluted with CHCl_3_–MeOH from 30:1 to 10:1 to afford PA (14 mg) and GA (15 mg). Fr.C3 (0.93 g) was isolated with silica gel column chromatography using CHCl_3_–MeOH from 30:1 to 2:1 as eluent to obtain TS (82 mg).

### 3.4. Cell Culture

The human colon adenocarcinoma cell line Caco-2 was purchased from the Cell Resource Center, Peking Union Medical College (CRC/PUMC, Beijing, China), and the Caco-2 cells between passages 32 and 36 were cultured in 25 cm^2^ flasks in DMEM medium supplemented with 10% (v/v) FBS, 1% (v/v) NEAA, 1% (v/v) penicillin-streptomycin, in an atmosphere of 5% CO_2_ and 95% air at 37 °C and constant humidity. The culture medium was replaced every 2–3 days, and the cells were split 1:2 or 1:3 when they reached 80% confluence.

### 3.5. MTT Assay

Caco-2 cells at logarithmic growth phase were seeded onto 96-well plates and incubated at 37 °C in culture hood. The culture medium was removed after cells formed a full layer, and each 100 μL of HBSS solution (negative control group) and drug-containing HBSS solution (three phenolic acids at the concentrations of 25, 50 and 100 μmol/L, respectively) was added to each well. When the plates were cultured for 4 h, HBSS solution was completely transferred out and each 100 μL of MTT solution (0.5 mg/mL) was added to each well. MTT solution was removed carefully after 4 hours of incubation at 37 °C, and each 150 μL of DMSO was added to each well, then culture vessels were agitated on an orbital shaker for 10 min and absorbance was read on a microplate reader at 570 nm.

### 3.6. Cell Differentiation

To facilitate the cells’ differentiation and formation of a confluent monolayer, Caco-2 cells were seeded at a density of 1.0 × 10^5^ cells/cm^2^ on a 12-well Transwell insert filter. The inserts were fed with culture medium at 2 days intervals in the first week, and then at daily intervals for the AP side and 2 days intervals for the BL side until they were used for the transport experiment 21 days after seeding. The AP and BL sides contained 0.5 mL and 1.5 mL of culture medium, respectively. The integrity and viability of the cell monolayers were evaluated by measuring TEER values in culture medium at 37 °C using Millicell^®^-ERS system (Millipore Corp., Bedford, MA, USA) and transport experiment using standard compounds, *i.e.*, propranolol and atenolol which were well-known control substances for high and poor transcellular transport markers, respectively. The cell inserts were used after the resistance reached above 600 Ω∙cm^2^.

### 3.7. Transport Experiment

On day 21, transport study was initiated by careful removal of the culture medium from AP and BL sides. Caco-2 monolayers were rinsed twice with pre-warmed HBSS and were incubated by pre-warmed HBSS for 30 min at 37 °C. PA, GA and TS were dissolved in DMSO, and diluted to the concentration of 25, 50 and 100 μmol/L with HBSS. The final DMSO concentration was less than 1%. Test compounds were added to the AP side (0.5 mL) or BL side (1.5 mL) of the inserts, while the receiving chamber contained the corresponding volume of HBSS. Standard markers (propranolol and atenolol) were just added to the AP side (0.5 mL). Incubation was performed at 37 °C for 180 min, with shaking at 50 rpm. At 30, 60, 90, 120, 150 and 180 min, 0.2 mL of solutions from BL side or AP side were collected, and replaced with an equal volume of HBSS. Samples were frozen immediately and stored below −20 °C before analysis. Upon completion of all permeation experiments, TEER values were measured to ensure that cell monolayer integrity and viability had not been adversely affected by experimental conditions.

### 3.8. HPLC Analysis

#### 3.8.1. Chromatographic Condition

PA, GA and TS were determined on Waters 1500 system consisting of a 1525 Binary HPLC pump, a 2489 UV/Visible detector, an on-line degasser and a manual injector connecting a 20 μL loop. The signals were acquired and processed using Windows XP-based Waters Breeze 2 software. HPLC analysis was performed on a Kromasil C_18_ ODS column (250 mm × 4.60 mm i.d., 5 μm particle size) with a guard column. For PA and GA, the mobile phase consisted of acetonitrile and 1% acetic acid (50:50, v/v) at a flow rate of 1.00 mL/min, UV detector was set at 269 nm and 251 nm respectively, and temperature was 35 °C. For TS, the mobile phase consisted of acetonitrile and 1% acetic acid (30:70, v/v) at a flow rate of 1.00 mL/min, UV detector was set at 251 nm and temperature was 35 °C. Each 20 μL of sample was injected into the HPLC after filtered through 0.45 μm Millipore filters. The chromatograms of the three phenolic acids are shown in Figure 2.

#### 3.8.2. Linearity, Precision and Stability

The calibration curves were constructed by plotting peak area (Y, mAU*min) *vs.* amount (X, in μmol). The linear equation of three phenolic acids were as follows: for PA, Y = 1.02 × 10^8^X + 484 (r = 0.9999) with a good linearity over the range from 2.5 × 10^−5^ μmol to 2.0 × 10^−4^ μmol; for GA, Y = 1.57 × 10^8^X + 372 (r = 0.9994) with a good linearity over the range from 1.25 × 10^−5^ μmol to 5.0 × 10^−4^ μmol; for TS, Y = 3.13 × 10^8^X − 271 (r = 0.9996) with a good linearity over the range from 1.0 × 10^−6^ μmol to 2.5 × 10^−4^ μmol. Quantification was carried out by peak area measurements in comparison with the calibration curves.

Precisions were determined by investigating the reference solutions of PA, GA and TS in sextuplicates during a single day as intra-day precision and duplicating the intra-day experiment on two consecutive days as inter-day precision. The RSD values of intra-day precision for the three analytes were 0.57%, 1.20% and 1.58%, respectively, while those values of inter-day precision for the three analytes were 0.62%, 1.19% and 2.17%, respectively. Each one sample solution of PA, GA and TS was kept at room temperature, and then the stability was determined by injecting it into apparatus at 0, 1, 2, 4, 6, 12 and 24 h. The RSD values for 24-hour stability of the three analytes were 1.69%, 2.03% and 2.26%, respectively. It demonstrated that the samples were stable within 24 h.

### 3.9. Calculation of Molecular Properties

The logarithm of octanol-water partition coefficient (logP) was assessed with ChemOffice 2004 (CambridgeSoft Corporation, Cambridge, MA, USA), and the other properties, such as TPSA, H_d_ (the number of hydrogen bond donor), H_r_ (the number of hydrogen bond receptor), R (the number of rotatable bond), and V (the number of violation of the “five rules”), were acquired from online products of Molinspiration Corporation. The results are listed in Table 3.

### 3.10. Data Analysis

The cell survival rate (%) was calculated using the equation survival rate= A/A_0_ × 100, wherein A was the average absorbance value of treatment group; A_0_ was the average absorbance value of the blank control group. The P_app_ was determined in this study by HPLC quantification of the compounds in the receiver chamber after transport across the Caco-2 monolayer. The calculation was described by the equation P_app_ = (dQ/dt) × (1/A) × (1/C_0_), wherein dQ/dt was the rate of appearance of the test compound on the receiver compartment (μmol/s); C_0_ was the initial test compound concentration on the donor compartment (μmol/mL); and A was the surface area of Caco-2 monolayer (cm^2^).

The percentage transported (%) was calculated using the equation % Transported = Q_B_/(C_S_ × V_D_) × 100, wherein Q_B_ was the amount of compounds in the receiver compartment(μmol); C_S_ was the donor concentration of test compounds (μmol/L); V_D_ was the volume of the donor compartment (L). The transport rate (μmol/h/cm^2^) was calculated using the equation transport rate = Q_B_/(t × A), wherein Q_B_ was the amount of compounds in the receiver compartment(μmol); t was the incubating time (h); A was the surface area of Caco-2 monolayer (cm^2^).

The results presented in this study were the averages of at least three replicates and were presented as means ± SD. The data were analyzed by either *t* test or nonparametric test after analysis of variance using SPSS 16.0. The level of significance was set at *p* < 0.05.

## 4. Conclusions

In summary, the present results provided a basis for the establishment of the major contributors to the efficacy of the flowers of *T. chinensis* and supplied useful information to predict the oral bioavailability of these phenolic acids.

## References

[1] Wang R.F., Yang X.W., Ma C.M., Liu H.Y., Shang M.Y., Zhang Q.Y., Cai S.Q., Park J. (2004). Trollioside, a new compound from the flowers of *Trollius chinensis*. J. Asian Nat. Prod. Res..

[2] Liu Y., Wang R.F., Yuan M., An Y.N., Wu X.W. (2012). HPLC Assay of trollioside in flowers of *Trollius chinensis*. Chin. J. Exp. Tradit. Med. Formulae.

[3] Yuan M., Wang R.F., Wu X.W., An Y.N., Yang X. (2013). Investigation on Flos Trollii: Constituents and bioactivities. Chin. J. Nat. Med..

[4] Li Y.L., Ma S.C., Yang Y.T., Ye S.M., But P.P. (2002). Antiviral activities of flavonoids and organic acid from *Trollius chinensis* Bunge. J. Ethnopharmacol..

[5] Yuan M.  (2013). The Transformation Production of the Constituents in the Flowers of *Trollius Chinensis* by Human Intestinal Bacteria and Their Antibacterial Activity. Master thesis.

[6] Li Q.F., Feng S.Q., Li Y.L., Cen Y.Z., Yang Y.T., Wang L.Y.  (2004). Study on the antibacterial and antiviral activity compositions of *Trollius chinensis* Bunge. J. Zhejiang Univ. (Sci. Ed.).

[7] Wang R.F. (2003). The Constituents and Bioactivities of Jinlianhua and Pangdahai. Doctoral Thesis.

[8] Yang X.W., Yang X.D., Wang Y., Ma L., Zhang Y., Yang X.G., Wang K. (2007). Establishment of Caco-2 cell monolayer model and standard operation procedure for assessing intestinal absorption of chemical components of traditional Chinese medicine. J. Chin. Integr. Med..

[9] Artursson P., Karlsson J. (1991). Correlation between oral drug absorption in humans and apparent drug permeability coefficients in human intestinal epithelial (Caco-2) cells. Biochem. Biophys. Res. Commun..

[10] Yee S.Y. (1997). *In vitro* permeability across Caco-2 cells (colonic) can predict *in vivo* (small intestinal) absorption in man-fact or myth. Pharm. Res..

[11] Kerns E.H., Di L. (2011). Drug-like Properties: Concepts,Structural Design and Methods.

